# The Prognosis Value of PIWIL1 and PIWIL2 Expression in Pancreatic Cancer

**DOI:** 10.3390/jcm8091275

**Published:** 2019-08-22

**Authors:** Weiyao Li, Javier Martinez-Useros, Nuria Garcia-Carbonero, Maria J. Fernandez-Aceñero, Luis Ortega-Medina, Sandra Garcia-Botella, Elia Perez-Aguirre, Luis Diez-Valladares, Jesus Garcia-Foncillas

**Affiliations:** 1 Translational Oncology Division, OncoHealth Institute, Fundacion Jimenez Diaz University Hospital, Av. Reyes Católicos 2, 28040 Madrid, Spain; 2 Pathology Department, University Hospital Gregorio Marañon, C/del Dr. Esquerdo 46, 28007 Madrid, Spain; 3 Pathology Department, Clinico San Carlos University Hospital, C/Profesor Martin Lagos, 28040 Madrid, Spain; 4 Surgery Department (Pancreatobiliary Unit), Hospital Clínico San Carlos, C/Profesor Martin Lagos, 28040 Madrid, Spain

**Keywords:** PIWI proteins, PIWIL1, PIWIL2, pancreatic cancer, prognostic biomarker, molecular subtypes

## Abstract

Pancreatic cancer is a highly aggressive manifestation of cancer, and currently presents poor clinical outcome due to its late diagnosis with metastasic disease. Surgery is the only approach with a curative intend; however, the survival rates seen in this type of patient are still low. After surgery, there is a lack of predictive prognosis biomarkers to predict treatment response and survival to establish a personalized medicine. Human P-element-induced wimpy testis 1 (PIWIL1) and P-element-induced wimpy testis 2 (PIWIL2) proteins act as protectors of germline, and their aberrant expression has been described in several types of tumors. In this study, we aimed to assess an association between PIWIL1 and PIWIL2 expression and the prognosis of biliopancreatic cancer patients. For this, we analyzed protein expression in complete resected tumor samples, and found a significant association between PIWIL2 expression and both progression-free and overall survival (*p* = 0.036 and *p* = 0.012, respectively). However, PIWIL2 expression was significantly associated with progression-free survival (*p* = 0.029), and overall survival (*p* = 0.025) of such tumors originated in the pancreas, but not in the bile duct or ampulla of Vater. Further analysis revealed that PIWIL1 and PIWIL2, at both mRNA and protein expression levels, correlated positively with factors associated to the progenitor molecular subtype of pancreatic cancer. Based on these findings, PIWIL1 and PIWIL2 expression may be considered a potential prognostic biomarker for resectable pancreatic cancer and may serve to guide subsequent adjuvant treatment decisions.

## 1. Introduction

Pancreatic cancer (PC) is one of the tumors with higher incidence in developed countries [1]. It is the fourth leading cause of cancer death in both sexes in the USA, and incidence continues to increase. Around 56,770 new cases of PC are estimated in the USA in 2019 (29,940 cases in men and 26,830 in women), and 45,750 deaths are estimated in the USA in 2019 (23,800 in men and 21,950 in women) [2]. PC is the eighth leading cause of cancer deaths in men and the ninth in women worldwide [3]. Indeed, the incidence of PC is expected to surpass breast, prostate, and colorectal cancers to become the second cause of cancer-related death by 2030 [4]. Although the five-year survival rate is 50% when tumors are <2 cm in size and close to 100% for tumors <1 cm [5], PC is normally asymptomatic, and it is often diagnosed at metastasic stages [6]. This fact drastically reduces patient survival to 3% of patients [2,7]. Ampullary adenocarcinoma is considered the tumor with the best prognosis of the biliopancreatic region. It is a relatively uncommon tumor and represents 0.2% of all digestive tumors [8]. The long-term prognosis is variable with survivals ranging between 37–75% at five years [9]. Regarding bile duct carcinomas, they present very low incidence (1–2 per 100,000 population) [10], and differentiation from ampullary adenocarcinoma is not easy to perform. Median survival of tumors originated in the bile duct is around 29 months, and the five-year survival rate is 27% [11]. To date, surgical resection with pancreatoduodenectomy (Whipple procedure) is considered the best procedure to manage tumors originated in the ampulla of the Vater, bile duct, or head of the pancreas. When tumors are localized in the tail or body of the pancreas, a distal pancreatectomy is performed and some cases required total pancreatectomy. Adjuvant treatment for complete resected patients (R0) is based on gemcitabine (1000 mg/m^2^ day 1, 8, 15/28 days) for six months [12], or 5-fluorouracil (425 mg/m^2^ and folinic acid 20 mg/m^2^ day 1–5 every 28 days) for six months [13]. The combination of gemcitabine (1000 mg/m^2^ day 1, 8, 15/28 days) and capecitabine (1660 mg/m^2^/day 1 to 21/28 days) for six months presented longer survival [14]. Regimens based on FOLFIRINOX or gemcitabine in combination with albumin-bound paclitaxel are recommended to patients with borderline resectable lesions [15]. Chemoradiotherapy is another option for borderline resectable patients with microscopically positive margin of resection (R1), and locally advanced unresectable disease [16,17].

Patient’s prognosis after resection could be predicted based on pathological parameters such as positive margins of resection, differentiation of tumor cells, lymph node status, etc. [18]. Therefore, an early detection of this type of cancer is crucial for successful treatment and to increase patient survival [19]. However, the only biomarker approved by the Food and Drug Administration (FDA) in PC is CA19-9 [20]. CA19-9 presents low specificity, so its utility has been questioned and its use is limited to predict recurrence after surgical resection [21]. Then, to better understand the poor prognosis of PC, further molecular studies are required [18].

P-element-induced wimpy testis (PIWI) proteins belong to the Argonaute (AGO) family and are expressed mainly in germline cells [22]. AGO proteins play an important role in the regulation of gene expression through complementary recognition of short RNAs, which guide them against their target genes [23]. In human, PIWI proteins consist of four members: PIWIL1, PIWIL2, PIWIL3, and PIWIL4 [24]. Specifically, PIWI proteins recognize and bind a type of non-coding small RNAs called piRNAs (PIWI-interacting RNAs), that constitutes the piRNA-induced silencing complex (piRISC). They have important roles in epigenetic regulation, the silencing of transposable elements, the protection of genome integrity, gametogenesis, and piRNA biogenesis [25].

In recent years, PIWI proteins have been linked to some of the hallmarks of cancer such as cell proliferation, the maintenance of genomic integrity, apoptosis evading, invasion, and metastasis [26,27]. This suggests that they could be used for cancer diagnosis and prognosis. The number of studies that show different expression patterns in healthy and tumor samples is increasing. In this context, an aberrant expression of PIWIL1 and PIWIL2 have been associated with different types of cancer and showed a variable prognostic and diagnostic potential [28]. The prognostic potential of PIWIL1 expression was previously evaluated in 56 PC samples at mRNA and protein levels. This study showed no impact on the survival of elevated PIWIL1 protein nor mRNA expression levels. However, altered mRNA expression (low or high expression compared to intermediate expression) presented poor prognosis only in the male population (*p* = 0.034) [29]. In respect to PIWIL2, the functional and clinical significance has not been reported in PC patients. Thus, the purpose of the present study is to evaluate the protein expression profile of PIWIL1 and PIWIL2 and assess the prognostic significance of these biomarkers in complete resected biliopancreatic tumors to guide subsequent adjuvant treatment decisions.

## 2. Experimental Section

### 2.1. Patients

A total of 190 biliopancreatic cancer patients who underwent surgery from 2006 to 2012 at the Surgery Department of University Hospital Clinico San Carlos were assessed for eligibility. Patients were followed-up to March 2019. Tumors were surgically resected and formalin-fixed and paraffin-embedded (FFPE) immediately for pathologic diagnosis. Tissue microarrays (TMA) were constructed with 182 available FFPE tumor samples. All the patients that presented positive margins of resection (R1) were excluded from the study (*n* = 53), resulting in 129 complete resected patients (R0). To assess survival analysis, only patients with available data of progression-free (*n* = 114) or overall survival (*n* = 117) were included in the study. At the end of the study, 45/114 (39%) patients did not progress, while 69/114 (61%) progressed on disease. Furthermore, 21/117 (18%) were alive, while 96/117 (82%) died at the study end. The tumor histology was reviewed by experienced pathologists. Since it is a retrospective study, PIWIL1 and PIWIL2 did not affect clinical decisions.

### 2.2. Immunohistochemistry

A tissue microarray was constructed for immunohistochemistry analysis and contained 364 cores (two cores per patient) using the MTA-1 tissue arrayer (Beecher Instruments, Tartu, Estonia). Each core (diameter, 1 mm) was punched from pre-selected tumor regions in paraffin-embedded tissues. Staining was conducted in 2-μm sections. Slides were deparaffinized by incubation at 60 °C for 10 min and incubated with PT-Link (Dako, Denmark) for 20 min at 95 °C in a high pH-buffered solution. To block endogenous peroxidase, holders were incubated with peroxidase blocking reagent (Dako, Denmark). Biopsies were incubated for 20 min with a 1:100 dilution of anti-PIWIL1 antibody (ab12337; Abcam, Cambridge, UK), 1:250 dilution of anti-PIWIL2 antibody (ab181340; Abcam, Cambridge, UK), 1:100 dilution of anti-hepatocyte nuclear factor (HNF)-4-alpha antibody (ab92378; Abcam Cambridge, UK), 1:20 dilution of anti-Mucin-17 (MUC17) antibody (ab122184; Abcam, Cambridge, UK), or 1:500 dilution of anti-pancreatic and duodenal homeobox 1 (PDX1) antibody (ab134150; Abcam, Cambridge, UK). Tissues were incubated with the appropriate anti-immunoglobulin horseradish peroxidase-conjugated polymer (EnVision, Dako, Denmark) to detect antigen–antibody reaction. All the antibodies and anti-Ig horseradish peroxidase-conjugated antibody presented high specificity, and no positiveness resulted from these antibodies individually. To determine immunohistochemistry conditions, different human tissues were used as a positive control according to The Human Protein Atlas (http://www.proteinatlas.org): Testis tissue for both anti-PIWIL1 and anti-PIWIL2 antibodies, small intestine tissue for the MUC17 antibody, human colon tissue for the HNF4A antibody, and human pancreatic tissue for the PDX1 antibody. Sections were then visualized with 3,3′-diaminobenzidine as a chromogen for 5 min and counterstained with haematoxylin. Photographs were taken with a stereo microscope (Leica DMi1, Wetzlar, Germany). To quantify the PIWIL1, PIWIL2, and MUC17 immunostaining, a semiquantitative HistoScore (Hscore) was calculated, and HNF4A and PDX1 immunostaining were categorized as positive or negative, since they are nuclear markers. The Hscore was determined by estimation of the percentage of positively stained cells with low, medium, or high intensity of staining, after applying a weighting factor to each estimate. The following formula was used: Hscore = (low%) × 1 + (medium%) × 2 + (high%) × 3, and the results ranged from 0 to 300. Quantification for each patient biopsy was calculated with the average of both cores by two independent researchers.

### 2.3. Statistical Analysis

The association between protein expression and survival, both progression-free and overall survival, was assessed with Kaplan–Meier curves, and analysis was performed with a log-rank test. Progression-free survival was defined as the interval between the dates of surgery and recurrence (local or distant). Overall survival was defined as the interval between the dates of surgery and patient death or lost follow-up. The best cut-off point to identify low-risk or high-risk patients was determined by ROC (Receiver Operating Characteristics) curves for both progression-free (Area under the curve (AUC) = 0.862 for PIWIL1; AUC = 0.801 for PIWIL2) and overall survival (AUC = 0.764 for PIWIL1; AUC = 0.840 for PIWIL2). The Cox proportional hazards model was used to assess the hazard ratios and confidence intervals of both PIWIL1 and PIWIL2 expression, and clinicopathological variables of patients with only pancreatic origin. Thus, only statistically significant variables found in the univariate analysis were included in the multivariate analysis.

The association between PIWIL1 or PIWIL2 expression and clinicopathological variables was evaluated by Chi-square or Fisher exact tests.

To describe the association between PIWIL1 and PIWIL2 mRNA and the most significant factors associated to each of the molecular profiles of pancreatic cancer described by Bailey et al. [30], a 186-patient dataset from The Cancer Genome Atlas (TCGA) was analyzed using cBioPortal [31,32]. To validate previous results at the protein level, the Kolmogorov–Smirnov test was used to determine the normal distribution of PIWIL1, PIWIL2, and MUC17 Hscores. A Spearman test was used to evaluate the linear correlation between non-parametric variables (PIWIL1, PIWIL2, and MUC17); interpretation was performed according to Cohen et al. [33]. Positive or negative nuclear staining of HNF4A or PDX1 were associated to PIWIL1 or PIWIL2 with the Chi-square test. *p*-value ≤ 0.05 was considered statistically significant. Statistical analysis was performed with the IBM SPSS program, version 20.0.

### 2.4. Ethics Statement

All the human samples were kindly supplied by the BioBank of University Hospital Clinico San Carlos (B.0000725; PT17/0015/0040; ISCIII-FEDER). All the patients gave written informed consent for the use of their biological samples for research purposes. The institutional review board (IRB) of the University Hospital Clinico San Carlos evaluated the present study, granting approval on 10 March 2017 with approval number nº 17/091-E. Moreover, fundamental ethical principles promoted by Spain (LOPD 15/1999) and the European Union Fundamental Rights of the EU (2000/C364/01) were followed. In addition, all the patients’ data were processed according to the Declaration of Helsinki (last revision 2013) and Spanish National Biomedical Research Law (14/2007, of 3 July).

## 3. Results

### 3.1. Patients Characteristics

Our cohort was well-balanced in terms of sex, and the median age of patients was 72 years (range 44 to 94 years). Pathologic diagnosis revealed the size of the resected tumors to be higher than 2 cm in 53% (*n* = 68) of cases. Tumors were stage I in 35% of cases (15% (*n* = 20) stage IA, and 20% (*n* = 26) stage IB); and stage II in 58% of cases (18% stage IIA (*n* = 23), and 40% (*n* = 51) stage IIB) according to the recommendations of the College of American Pathologists [34]. Most of the patients did not receive adjuvant treatment (59%, *n* = 76). Tumors presented as low grade in 82% (*n* = 106) of cases. Forty percent of patients (*n* = 51) showed lymph-node involvement, and most patients had vascular and neural invasion (33% (*n* = 43) and 55% (*n* = 71), respectively). Tumors were originated in the pancreas in 65% (*n* = 84), in the ampulla in 18% (*n* = 23), and in the bile duct in 15% (*n* = 20) of the cases. All the patients included in the study were completely resected, and thus presented negative surgical margins of resection (R0). An overview of the clinicopathological parameters of the patients is given in Table 1.

To verify whether the expression of PIWIL1 or PIWIL2 could be closely related to any of the clinicopathological characteristics registered in our study, a crosstab was performed thereafter (Table 2). In this analysis, PIWIL1 was associated significantly with gender (*p* = 0.035), where low levels of PIWIL1 are more often present in the male population, and also showed a high trend toward significance with pancreatic origin (*p* = 0.072). Low PIWIL2 expression had a statistically significant association with a higher T stage (*p* = 0.040). This result suggests that the lack of PIWIL2 expression exhibits a deleterious effect on the patients analyzed. Furthermore, lower PIWIL2 expression exhibited a trend toward significance with other pathologic characteristics associated to tumor aggressiveness such as vascular invasion (*p* = 0.068), neural invasion (*p* = 0.108), tumor stage (*p* = 0.111), or lymph nodes involved (*p* = 0.128) (Table 2).

### 3.2. PIWIL1 Expression has no Impact on Patient Survival

**Figure 1:**
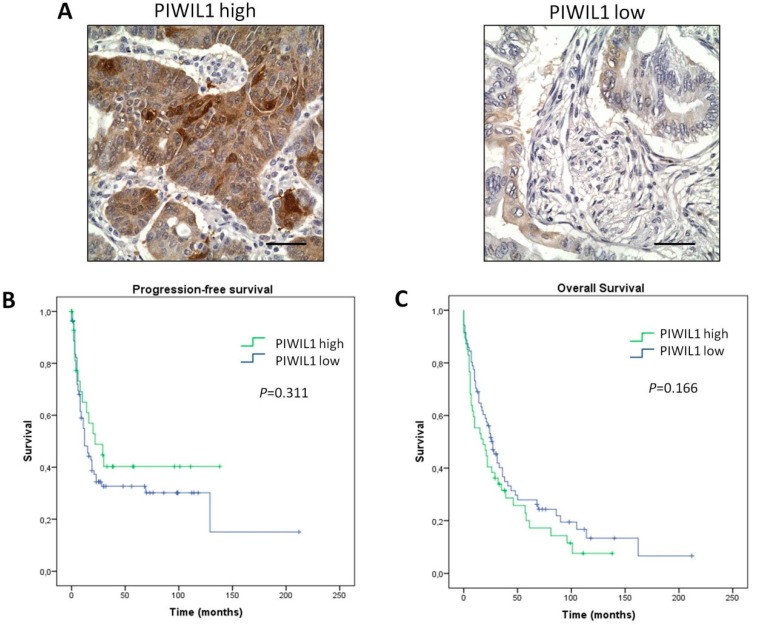
PIWIL1 expression has no impact on the outcome of biliopancreatic cancer patients. (**A**) Representative micrographs of high (left) and low (right) PIWIL1 expression tumors. (**B**,**C**) Kaplan–Meier curves for progression-free survival and overall survival analysis of patients, respectively. P-values were obtained by log-rank test. Scale bars: 50 μm.

All the samples that stained positively for PIWIL1 exhibited a cytoplasmic expression pattern and some membrane localization, especially in some cases with high expression levels (Figure 1A left). In fact, PIWIL1 expression was also detected in the cytoplasm of some stroma cells, although all the cases with positive stained stroma cells showed stronger positiveness in tumor cells than in the stroma. Subsequently, tumor samples were divided into low or high expression of the PIWIL1 protein according to the ROC curve to associate its expression to survival (Figure 1B,C). Association between PIWIL1 and both progression-free and overall survival did not achieve statistical significance (*p* = 0.311 and *p* = 0.166, respectively).

### 3.3. PIWIL2 Expression Associated with Better Prognosis of Patients

**Figure 2:**
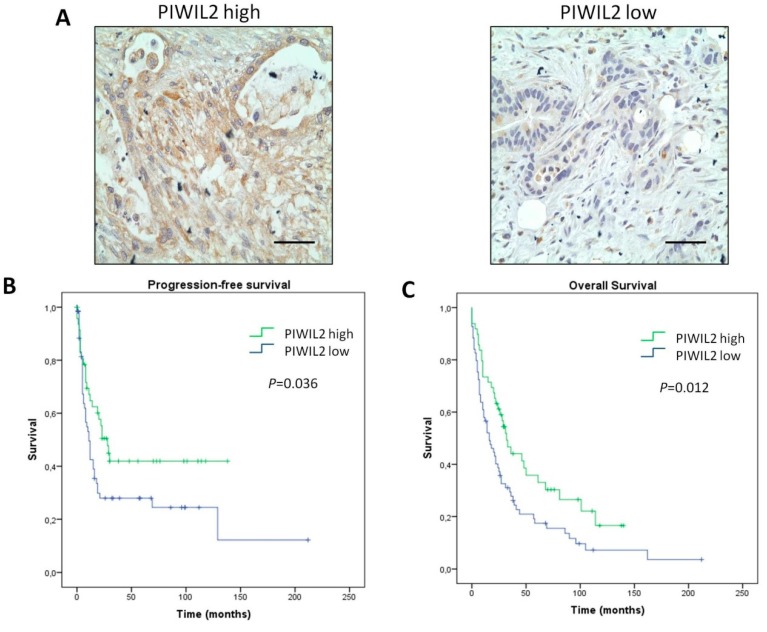
PIWIL2 expression predicts better prognosis in biliopancreatic cancer patients. (**A**) Representative micrographs of high-risk (left) and low-risk (right) PIWIL2 expression tumors. (**B**,**C**) Kaplan–Meier curves for progression-free survival and overall survival analysis of patients, respectively. P-values were obtained by log-rank test. Scale bars: 50 μm.

Since PIWIL2 protein expression has not yet been determined by The Human Protein Atlas Project, we used a testis sample as a control to test the optimum concentration of antibody, as previously performed for PIWIL1 evaluation (Figure S1). PIWIL2 protein expression not only localized on the cytoplasm of tumor cells, but also weakly in the cytoplasm of stroma cells in those cases with high PIWIL2 expression levels (Figure 2A left). The association between PIWIL2 protein expression and outcome of patients was assessed. For this, patients were stratified into low-risk and high-risk according to a cut-off point determined by the ROC curve. Interestingly, the expression of PIWIL2 protein in tumor samples had a statistically significant association with progression-free survival (*p* = 0.036; Figure 2B). Indeed, patients with a high expression of PIWIL2 presented longer median progression-free survival (median = 28 months; 95% CI: 18–38 months) than patients with low PIWIL2 expression (median = 11 months; 95% CI: 7–15 months). Then, an association between PIWIL2 protein expression and overall survival was also assessed. PIWIL2 protein expression was associated with longer overall survival (*p* = 0.012; Figure 2C). Here, patients with high PIWIL2 expression presented longer overall survival (median = 32 months; 95% CI: 23–41 months), while patients with low expression of PIWIL2 showed shorter overall survival (median = 16 months; 95% CI: 8–24 months). These results suggest that low PIWIL2 expression is a negative variable for survival outcome.

**Figure 3:**
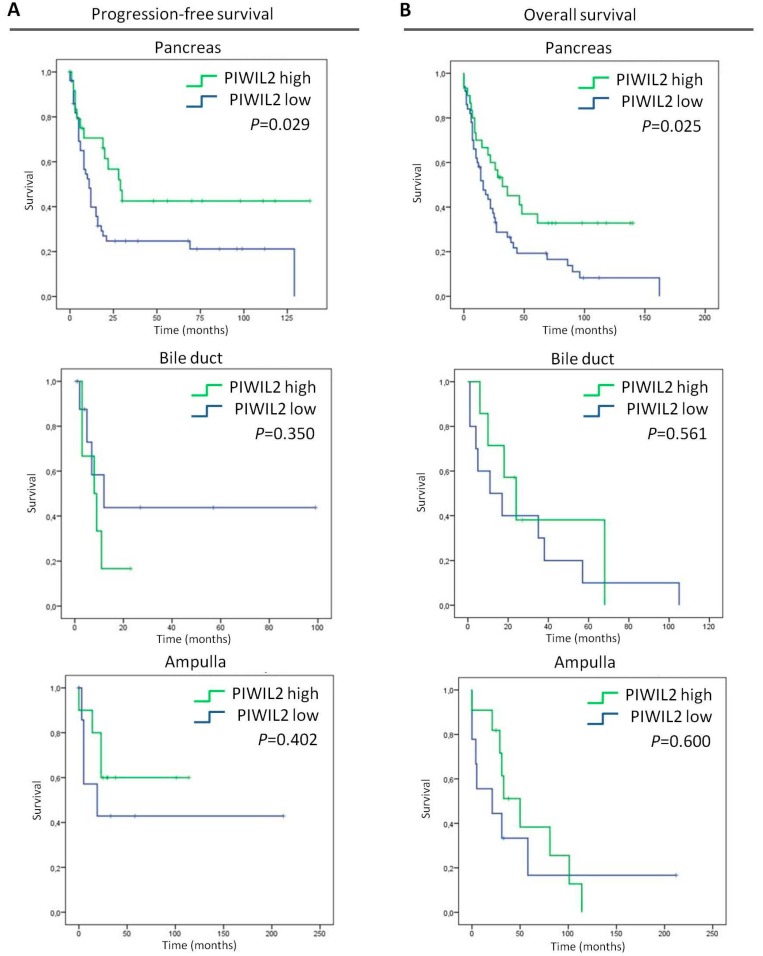
Survival analyses of patients according to their tumor origin and PIWIL2 expression. (**A**) Kaplan–Meier plots of progression-free survival, and (**B**) Kaplan–Meier plots of the overall survival of patients with a high expression of PIWIL2 (green lines) and patients with a low expression of PIWIL2 (blue lines). *p*-values were obtained by the log-rank test.

Since all the biliopancreatic tumors included in the present study originated in the pancreas (*n* = 84), in the bile duct (*n* = 20), or in the ampulla of Vater (*n* = 23), we analyzed both the progression-free and overall survival of patients according to PIWIL2 expression stratified by their tumor origin (Figure 3). Interestigly, PIWIL2 expression associated with pancreatic origin in both progression-free survival and overall survival. Patients with pancreatic tumor origin that exhibited a high expression of PIWIL2 presented longer median progression-free survival (median = 29 months; 95% CI: 17–40 months) than patients with low PIWIL2 expression (median = 11 months; 95% CI: 7–14 months) (*p* = 0.029; Figure 3A-top). The overall survival of patients with pancreatic tumor origin with high PIWIL2 expression was longer (median = 32 months; 95% CI: 8–55 months) than patients with low expression of PIWIL2 (median = 16 months; 95% CI: 8–23 months) (*p* = 0.025; Figure 3B-top). The other tumor origins such as bile duct or ampulla were not associated with PIWIL2 expression for neither progression-free (Figure 3A—middle and bottom, respectively) nor overall survival (Figure 3B—middle and bottom, respectively). Therefore, this result supports the role of PIWIL2 as a prognostic biomarker in those biliopancreatic tumors that originated in the pancreas.

In order to compare the potential prognosis value of PIWIL2 expression with the other clinical variables, we performed a Cox proportional hazards model for both progression-free and overall survival of patients with only pancreatic tumor origin (Table 3). The univariate analysis for progression-free survival revealed that patients with a low expression of PIWIL2 showed a higher risk of recurrence after surgery compared to those with a high expression of PIWIL2 (hazard ratio, or HR = 1.788; 95% CI: 0.987–3.249). Although PIWIL2 did not raise significance to predict progression-free survival, our research found a high trend toward significance (*p* = 0.057). Here, the only significant variable associated with progression-free survival was the tumor stage—especially stage IIA, which presented the highest risk (HR = 3.568; 95% CI: 1.221–10.431; *p* = 0.020). On the other hand, the univariate analysis for overall survival revealed the potential of low levels of PIWIL2 to predict poor prognosis (HR = 1.832; 95% CI: 1.064–3.154; *p* = 0.029). Moreover, neural invasion appeared to be statistically associated with overall survival (HR = 1.819; 95% CI: 1.000–3.310; *p* = 0.050). Then, multivariate analysis between PIWIL2 expression and neural invasion as covariate revealed PIWIL2 as the unique statistically significant factor associated to overall survival (HR = 1.726; 95% CI: 0.946–3.154; *p* = 0.039) (Table 3). Thus, this result highlights the role of low expression of PIWIL2 as a detrimental factor in tumors with pancreatic origin.

### 3.4. PIWIL1 and PIWIL2 Expression is Associated to Progenitor Molecular Subtype of Pancreatic Cancer

**Figure 4:**
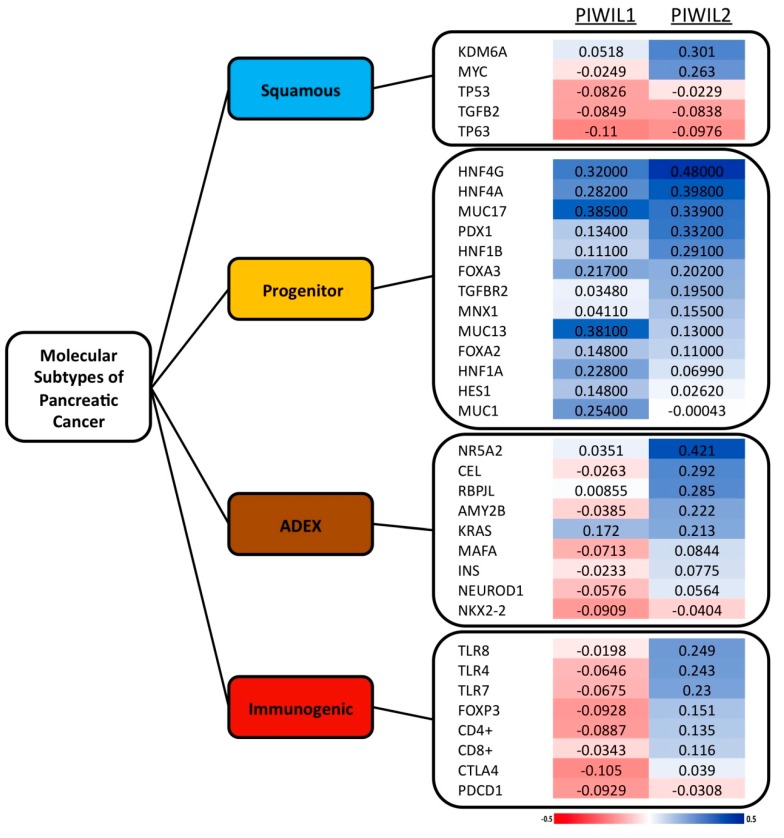
Expression of piwil1 or piwil2 is associated with most significant factors of the progenitor molecular subtype of pancreatic cancer at the mRNA level. The figure shows the Spearman correlation coefficients between the most relevant genes for each molecular subtype versus piwil1 or piwil2 mRNA expression. The red charts correspond to negative coefficients and the blue charts correspond to positive coefficients, in a scale ranging from −0.5 to 0.5.

Given our previous results related to patient outcome, we wonder whether PIWIL1 or PIWIL2 would be associated to any of the four described molecular subtypes of pancreatic cancer [30]. Then, mRNA expression of the most significant factors associated with each molecular subtype of pancreatic cancer of 186 pancreatic cancer patients from a TGCA dataset was correlated with PIWIL1 or PIWIL2 mRNA expression levels (Figure 4). Interestingly, we found a positive moderate correlation between PIWIL1 or PIWIL2 and most of the genes that characterize the progenitor molecular subtype. Here, PWIIL1 mRNA correlated moderately with MUC17 (r = 0.385), MUC13 (r = 0.381), HNF4g (r = 0.320), HNF4a (r = 0.282), MUC1 (r = 0.254) or HNF1a (r = 0.228). PIWIL2 mRNA exhibited a positive moderate correlation with HNF4g (r = 0.480), HNF4a (r = 0.398), MUC17 (r = 0.339), PDX1 (r = 0.332) or HNF1b (r = 0.291) (Figure 4).

Since these findings are observed at the mRNA level, we decided to validate this correlation at the protein level. We chose three different factors; within these, mRNA correlated with piwil1 and piwil2 with a higher correlation coefficient and that associated to different molecular mechanisms, according to three criteria. Firstly, as HNF4G is not expressed in pancreatic tissue according to The Human Protein Altas, we selected HNF4A (hepatocyte nuclear factor 4) because of its crucial role in pancreatic β-cells development and since its mutations cause a type of maturity-onset diabetes of the young. Secondly, we selected MUC17 (Mucin-17), which is a type of mucin overexpressed in pancreatic tumor cell lines and tumor tissues compared with the normal pancreas. Finally, we selected PDX1 (pancreatic and duodenal homeobox 1), which is necessary for pancreatic development, including β-cell maturation.

Consequently, we stained our 186 tumor cohort to evaluate these markers (Figure S2). Curiously, PIWIL2 protein expression correlated positively with MUC17 (r = 0.225; *p* = 0.002). Furthermore, PIWIL2 associated significantly with HNF4A (*p* = 0.016), and PIWIL1 associated significantly with PDX1 (*p* = 0.036). Hence, statistical analyses of both mRNA and the protein level support the association between PIWIL1 and PIWIL2 with the progenitor molecular subtype of PC.

## 4. Discussion

Despite all the scientific advances in PC, patients’ outcome is still poor, and incidence of this disease is increasing; therefore, we need new molecular targets to bring promising approaches. In this regard, PIWI proteins provide new insights to address the therapeutic challenges of PC. The *PIWI* gene family is a novel class of highly conserved genes that encodes basic proteins with a high homology [35]. PIWI proteins incorporate both a RNA endonuclease catalytic domain and an anchor site for the 5′ phosphate of the RNA guide strand [36], which confers their ability to recognize small interfering RNA known as PIWI-interacting RNAs [37,38,39]. PIWI proteins are involved in stem cell division, gametogenesis, germline specification, and RNA silencing [35,40,41]. In humans, PIWI proteins are necessary for spermatogenesis [35] and the maintenance of hematopoietic stem cells [42]. Therefore, the conserved functions of PIWI proteins in stem cell maintenance suggest their potential role in tumorigenesis with a low grade of differentiation. The first tumor where PIWI proteins were studied was a testis tumor that originated from germ and non-germ cells [43].

In this clinical research, we have focused on the study of PIWIL1 and PIWIL2 protein expression to dissect their prognostic value in PC and assist physicians in patient management. PIWI proteins have been associated to several neoplasias after being described for the first time in a testis tumor [26]. The *PIWIL1* gene is located closed to the extreme of the long arm of chromosome 12q24.33, and its expression was associated to gastric cancer and precancerous development with an expression pattern similar to Ki67 [44]. In addition, PIWIL1 has been described to exhibit a poor prognostic value in soft-tissue sarcoma [45,46], esophageal squamous cell carcinoma [47], colorectal cancer [48,49,50], gliomas [51], human hepatocellular carcinoma [52,53], gastric cancer [54,55], lung cancer [56], gynecological cancers [57,58,59], renal cell carcinoma [60,61], and non-small cell lung cancer [62]. In contrast, the overexpression of PIWIL1 seems to have a beneficial effect in chronic myeloid leukemia, since it is able to inhibit the growth and migration of tumor cells [63] and increases sensitivity to daunomycin [64]. Moreover, PIWIL1 is considered a potential target for treatment design in glioblastoma [65], hepatocellular carcinoma [66], and lung cancer [67]. Only one study reported the prognosis significance of PIWIL1 in PC determined by mRNA and protein levels [29]. As observed by authors, neither PIWIL1 protein nor mRNA expression levels had an impact on survival. However, in this study, the altered mRNA expression (high or low) presented shorter survival than those patients with intermediate levels of PIWIL1 mRNA (*p* = 0.034) [29]. From our point of view, intermediate mRNA expression should be rather limited at clinical practice. For this reason, we focused our study on protein expression levels evaluated by immunohistochemical staining. Similarly to previously reported results, PIWIL1 protein expression does not have enough statistical power to be considered a prognostic biomarker, although it exhibited an association with male patients and a trend toward significance with pancreatic tumor origin.

On the other hand, we have also evaluated the prognostic value of PIWIL2 in our patient cohort. The *PIWIL2* gene is localized in chromosome 8p21.3, and its expression has also been associated with tumor development of some gynecological cancers [57,59], renal cell carcinoma [60,61], breast cancer [68], gastric cancer [54], and colorectal cancer [69]. Since The Human Protein Atlas Project only provides PIWIL2 mRNA expression, we described for the first time the protein expression profile of PIWIL2 in human tumor samples by using human testis tissues to determine the best antibody concentration. Consequently, survival analyses in our patient cohort according to PIWIL2 protein expression revealed the lack of PIWIL2 as a negative event in prognosis that reduces both progression-free and overall survival. Furthermore, the survival analysis performed with patients stratified by tumor origin revealed the prognosis significance of PIWIL2 expression and its potential value to predict the outcome of patients with tumors that originated in the pancreas. This effect was also supported by the Cox multivariate analysis for overall survival, where PIWIL2 expression remained the only significant molecular event ahead of neural invasion. Moreover, the statistical association between low expression levels of PIWIL2 and higher T status, and a high trend toward significance with vascular invasion, neural invasion, and higher stages support the role of low expression levels of PIWIL2 as a detrimental effect on the progression and development of such tumors. Our result was in accordance with the findings of Litwin et al., which reported decreased PIWIL2 mRNA expression in colorectal cancer tissues compared to untransformed tissues (*p* < 0.001) [70]. PIWIL2 protein expression has also been found to be downregulated in non-small cell lung cancer samples in comparison to the normal tissue (*p* < 0.001) [62]. In addition, PIWIL2 mRNA levels have been statistically significant lower in breast carcinoma samples compared to normal breast tissues (*p* < 0.001) [70]. Consequently, the tumorigenic effect of PIWIL2 expression seems to be rather controversial and remains unclear. In this concern, the epigenetic modulation of PIWI proteins’ expression plays a crucial role and could justify their ambivalent role in tumorigenesis through the upregulation of DNA methyltransferases [71].

PIWI proteins regulate several molecular pathways through key mediators in different neoplasias. Here, we describe that most of the significant factors associated to the progenitor molecular subtype of PC have a positive correlation with PIWIL1 or PIWIL2 at the mRNA level. Moreover, the protein expression of PIWIL2 correlated positively with MUC17 and associated significantly with HNF4A at the protein level, while PIWIL1 expression associated significantly with PDX1 protein expression. This fact supports the link between these two PIWI proteins and the progenitor molecular subtype of PC, which is involved in early pancreatic endoderm development and related to maturity onset diabetes of the young [30].

Besides the link between PIWI proteins and progenitor subtype, these proteins have been associated to several genes involved in the cell cycle regulation, apoptosis, proliferation, and migration of tumor cells. For example, PIWIL1 is able to regulate OCT4, which is a factor associated to poor prognostic and metastasic disease in colorectal cancer [70]. PIWIL1 also regulates apoptosis and cell cycle progression through P21, Cyclin D1, BCL-2 and BAX, and migration through expression of MMP-2 and MMP-9 in glioma cells [72]. In contrast, PIWIL1 expression downregulates MMP-2 and MMP-9 and inhibits the proliferation and migration ability of chronic myeloid leukemia cells [63]. In gastric cancer, *PIWIL1* has been related to *OCK2*, *ZNF503*, *PDE4D*, *ABL1*, *ABL2*, *LPAR1*, *SMAD2*, *WASF3*, and *DACH1* genes [73], and it has exhibited a regulation activity of epithelial-to-mesenchymal transition in endometrial cancer [74]. PIWIL2 regulates BCL-XL and STAT3, and its downregulation both suppresses protein expression triggering apoptosis cascade [75], and enhances cisplatin sensitivity [76]. Interestingly, Chen et al. suggested that the ectopic expression of PIWIL2 contributes to the development and proliferation of precancerous stem cells, which have the potential for both benign and malignant differentiation [77]. Furthermore, a positive correlation between PIWIL2 and the undifferentiated cell marker SOX2 have been observed in colorectal cancer tissues [70].

## 5. Conclusions

This study shows the differential role of PIWIL1 and PIWIL2 in pancreatic cancer. On the one hand, the expression of PIWIL1 did not associate to pancreatic cancer prognosis; thus, further research is needed to dissect the role of PIWIL1 in pancreatic cancer progression. On the other hand, the results presented here support the role of PIWIL2 protein expression as a prognostic biomarker in pancreatic cancer, and suggest a link between PIWIL2 expression and the progenitor molecular subtype of pancreatic cancer. However, PIWIL2 function in cancer initiation and development is rather controversial, and remains unclear. Therefore, future translational research might be focused on those piRNAs regulated by PIWIL2. The identification of piRNAs, in both solid tumors and serum samples, and their function will provide new insights of PIWI proteins and their role as diagnostic, prognostic, or predictive biomarkers.

## Figures and Tables

**Table 1:** Clinicopathological characteristics of complete resected biliopancreatic cancer patients recruited in the study.

Clinical Characteristics	*n*	%	Clinical Characteristics	*n*	%
**Age**			**Grade**		
<65 years	25	19	High	19	15
>65 years	104	81	Low	106	82
**Gender**			N/A	4	3
Male	63	49	**Vascular invasion**		
Female	66	51	No	76	59
**Adjuvant treatment**			Yes	43	33
No	76	59	N/A	10	8
Yes	24	19	**Neural invasion**		
N/A	29	22	No	48	37
**Tumor origin**			Yes	71	55
Pancreas	84	65	N/A	10	8
Ampulla	23	18	**pT**		
Bile duct	20	15	T1	30	23
N/A	2	2	T2	45	35
**Size**			T3	51	40
<2 cm	31	24	N/A	3	2
>2 cm	69	54	**Lymph nodes involved**		
N/A	29	22	No	70	54
**Stage**			Yes	51	40
IA	20	15	N/A	8	6
IB	26	20	**TOTAL**	129	100
IIA	23	18			
IIB	51	40			
N/A	9	7			

N: number of patients; N/A: Not available; cm: centimeters.

**Table 2:** Statistical association between P-element-induced wimpy testis 1 (PIWIL1) and P-element-induced wimpy testis 2 (PIWIL2) protein expression with clinico-pathological characteristics.

	PIWIL1 Low	PIWIL1 High		PIWIL2 Low	PIWIL2 High	
Parameters	*n* (%)	*n* (%)	*p*-Value	*n* (%)	*n* (%)	*p*-Value
**Gender**			0.035			0.390
Male	43 (33%)	20 (15%)		40 (31%)	23 (18%)	
Female	33 (26%)	33 (26%)		37 (29%)	29 (22%)	
**Age**			0.304			0.383
<65 years	17 (13%)	8 (6%)		13 (10%)	12 (9%)	
>65 years	59 (46%)	45 (35%)		64 (50%)	40 (31%)	
**Stage**			0.560			0.111
IA	9 (8%)	11 (9%)		7 (6%)	13 (11%)	
IB	17 (14%)	9 (8%)		15 (12%)	11 (9%)	
IIA	14 (11%)	9 (8%)		14 (12%)	9 (8%)	
IIB	30 (25%)	21 (17%)		34 (28%)	17 (14%)	
**Adjuvant treatment**			0.324			0.689
No	42 (42%)	34 (34%)		44 (44%)	32 (32%)	
Yes	16 (16%)	8 (8%)		15 (15%)	9 (9%)	
**pT**			0.960			0.040
T1	17 (13%)	13 (10%)		12 (10%)	18 (14%)	
T2	26 (21%)	19 (15%)		27 (21%)	18 (14%)	
T3	28 (22%)	23 (18%)		35 (28%)	16 (13%)	
**Size**			0.536			0.968
<2 cm	15 (15%)	16 (16%)		19 (19%)	12 (12%)	
>2 cm	38 (38%)	31 (31%)		42 (42%)	27 (27%)	
**Lymph nodes involved**			0.978			0.128
No	41 (34%)	29 (24%)		37 (31%)	33 (27)	
Yes	30 (25%)	21 (17%)		34 (28%)	17 (14%)	
**Vascular Invasion**			0.893			0.068
No	45 (38%)	31 (26%)		40 (34%)	36 (30%)	
Yes	26 (22%)	17 (14%)		30 (25%)	13 (11%)	
**Neural Invasion**			0.891			0.108
No	29 (24%)	19 (16%)		24 (20%)	24 (20%)	
Yes	42 (35%)	29 (25%)		46 (39%)	25 (21%)	
**Grade**			0.142			0.703
Low	59 (47%)	47 (38%)		62 (50%)	44 (35%)	
High	14 (11%)	5 (4%)		12 (10%)	7 (5%)	
**Tumor origin**			0.072			0.197
Pancreas	67 (53%)	17 (13%)		54 (43%)	30 (24%)	
Bile duct	14 (11%)	6 (5%)		12 (9%)	8 (6%)	
Ampulla	13 (10%)	10 (8%)		10 (8%)	13 (10%)	

N: Number of patients; cm: centimeters.

**Table 3:** Univariate and multivariate proportional hazard model of PIWIL2 and other clinical variables for progression-free and overall survival of those patients with pancreatic tumor origin.

	Univariate PFS (95% CI)				Univariate OS (95% CI)			
	HR	Lower	Upper	*p-*Value	HR	Lower	Upper	*p-*Value
**Age** (<65 years vs. >65 years)	1.359	0.695	2.657	0.370	1.320	0.715	2.439	0.375
**Gender** (Male vs. Female)	1.420	0.814	2.478	0.217	1.083	0.657	1.785	0.756
**Adjuvant treatment** (Yes vs. No)	1.168	0.603	2.260	0.645	1.321	0.781	2.428	0.371
**Size** (<2 cm vs. >2 cm)	1.792	0.633	5.073	0.272	1.163	0.519	2.603	0.714
**Stage**				0.050				0.148
IA	1.000				1.000			
IB	1.367	0.466	4.008	0.569	1.161	0.466	2.890	0.748
IIA	3.568	1.221	10.431	0.020	2.491	0.975	6.362	0.056
IIB	2.393	0.900	6.364	0.080	1.733	0.743	4.040	0.203
**Grade** (low vs. high)	1.156	0.492	2.716	0.740	1.138	0.540	2.395	0.734
**Lymph nodes involved** (No vs. Yes)	1.457	0.817	2.599	0.202	1.266	0.746	2.150	0.382
**Vascular invasion** (No vs. Yes)	1.466	0.809	2.658	0.208	1.427	0.827	2.464	0.202
**Neural invasion** (No vs. Yes)	1.815	0.936	3.521	0.078	1.819	1.000	3.310	0.050
**pT** (I vs. II/III)	1.690	0.841	3.396	0.141	1.505	0.797	2.841	0.207
**PIWIL2** (high vs. low)	1.788	0.987	3.249	0.057	1.832	1.064	3.154	0.029
	**Multivariate PFS (95% CI)**				**Multivariate OS (95% CI)**			
**Neural invasion** (No vs. Yes)					1.726	0.946	3.151	0.075
**PIWIL2** (high vs. low)					1.813	1.030	3.189	0.039

PFS: progression-free survival; OS: Overall survival; HR: hazard ratio; CI: confidence interval; vs.: versus; cm: centimeters.

## Supplementary Materials

The following are available online at https://www.mdpi.com/2077-0383/8/9/1275/s1, Figure S1: Immunohistochemical staining for PIWIL1 and PIWIL2 protein expression at different antibodies concentrations in human testis tissue; Figure S2: Immunohistochemical staining for HNF4A, MUC17 and PDX1 protein expression.
