# Supplementary material for: The Prognosis Value of PIWIL1 and PIWIL2 Expression in Pancreatic Cancer
Source: J Clin Med. 2019 Aug 22;8(9):1275. doi: 10.3390/jcm8091275 (PMC6780139; doi:10.3390/jcm8091275)
Supplement: Supplementary file 1 [file jcm-08-01275-s001.pdf]

Journal of
Clinical Medicine

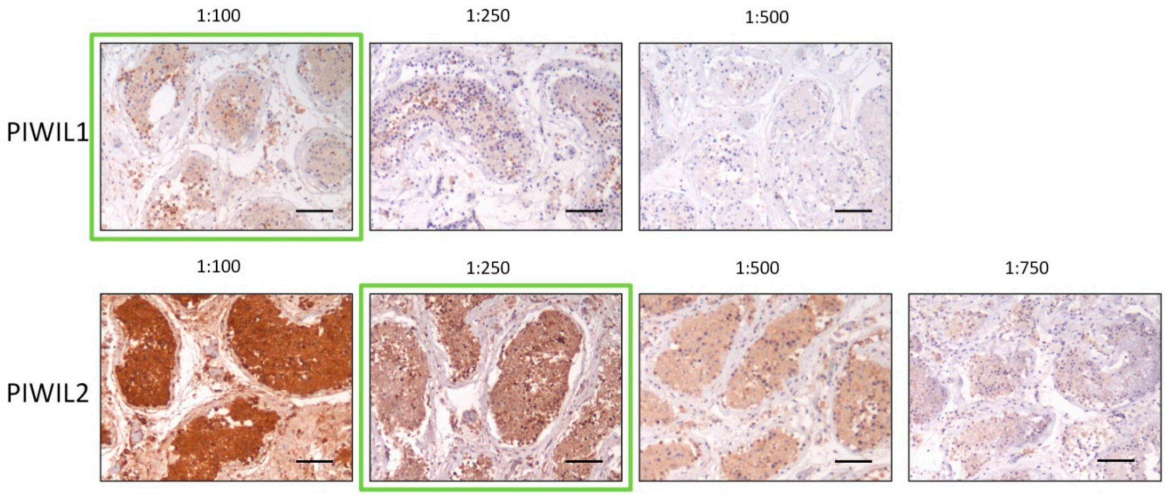

Figure S1. Immunohistochemical staining for PIWIL1 and PIWIL2 protein expression at different antibodies concentrations in human testis tissue. 1:100, 1:250, and 1:500 dilutions for anti-PIWIL1 antibody; and 1:100, 1:250, 1:500, and 1:750 dilutions for anti-PIWIL2 antibody were assessed. Green boxes show the optimal working dilution for each antibody. Scale bars represent 100 µm.

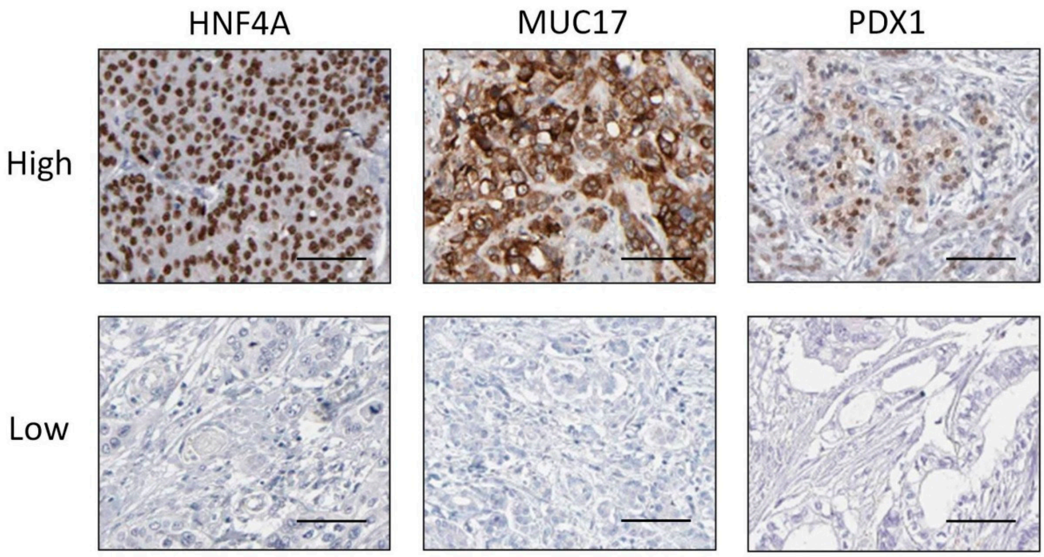

Figure S2. Immunohistochemical staining for hepatocyte nuclear factor 4A (HNF4A), Mucin-17 (MUC17), and pancreatic and duodenal homeobox 1 (PDX1) protein expression. The figure shows representative images of high (up) and low (down) expression tumors for each antibody. Scale bars represent 50 µm.
